# Diagnostic Utility of Monocyte Distribution Width for Early Sepsis Detection in Cancer-Enriched Emergency Cohort

**DOI:** 10.3390/jcm14228089

**Published:** 2025-11-14

**Authors:** Yong Jun Choi, Jooheon Park, Ha Jin Lim, Yong Jun Kwon, Hyun-Woo Choi, Seung-Jung Kee, Soo Hyun Kim, Myung Geun Shin, Eun-Hee Nah, Jong Hee Shin

**Affiliations:** 1 Department of Laboratory Medicine, Chonnam National University Hwasun Hospital, Hwasun 58128, Republic of Korea; azarsis@hanmail.net (Y.J.C.); alpinboy@hanmail.net (S.H.K.); mgshin@chonnam.ac.kr (M.G.S.); 2 Department of Laboratory Medicine, Chonnam National University Hospital, Gwangju 61469, Republic of Korea; hajin00905@naver.com (H.J.L.); garei09@gmail.com (Y.J.K.); wiseltree@gmail.com (H.-W.C.); sjkee1968@naver.com (S.-J.K.); cellonah@hanmail.net (E.-H.N.); shinjh@chonnam.ac.kr (J.H.S.)

**Keywords:** monocyte distribution width, diagnostic performance, sepsis, cancer patients, emergency department

## Abstract

**Background**: Timely recognition of sepsis remains a critical clinical challenge, particularly in cancer patients, who are at higher risk due to immunosuppression. Monocyte distribution width (MDW) has emerged as a biomarker with potential utility in the early detection of sepsis. **Methods**: This retrospective study analyzed 1167 patients who presented to the emergency department of a cancer specialty hospital in Republic of Korea. Patients were classified according to Sepsis-2 and Sepsis-3 criteria, and the diagnostic performance of MDW was compared with conventional biomarkers, including C-reactive protein (CRP) and procalcitonin (PCT). Subgroup analyses were conducted based on malignancy status, leukopenia, and initial signs of infection. Additionally, turnaround times (TATs) were compared among the biomarkers. **Results**: MDW demonstrated diagnostic accuracy comparable to or exceeding that of CRP and PCT for identifying sepsis and infection across both Sepsis-2 and Sepsis-3 criteria. In the context of diagnosing sepsis using the Sepsis-3 criteria, MDW yielded the highest area under the curve (0.869), sensitivity (91.0%), and negative predictive value (98%). Notably, in cancer patients, MDW maintained strong diagnostic reliability. It also demonstrated high diagnostic capability in patients with leukopenia or presenting with initial signs of infection. Moreover, the TAT was significantly shorter for MDW (median 59 min) than for CRP (105 min) or PCT (111 min). **Conclusions**: MDW is a rapid and accessible biomarker with demonstrated value for early sepsis detection in emergency settings. Its balanced diagnostic profile and consistent performance across diverse patient subgroups support its integration into routine clinical workflows, especially as part of multimodal sepsis screening strategies.

## 1. Introduction

Sepsis is defined as life-threatening organ dysfunction caused by a dysregulated host response to infection [1], which can ultimately lead to death. Early diagnosis is crucial, as it significantly improves the chances of survival through timely and appropriate treatment [2,3]. Therefore, healthcare providers must be vigilant in recognizing the early signs and symptoms of sepsis to initiate prompt intervention.

Cancer patients are particularly vulnerable to sepsis due to their weakened immune systems, either from the malignancy itself or as a consequence of immunosuppressive therapies such as chemotherapy and radiotherapy [4]. These patients face a tenfold greater risk of sepsis compared to those without cancer, with notable differences across cancer subtypes [5].

Monocyte distribution width (MDW) has emerged as a promising biomarker for the early diagnosis of sepsis [6,7]. MDW is readily available through a routine complete blood count with differential (CBC-DIFF), providing a rapid and accessible indicator. When the immune system responds to infection, monocytes become activated and exhibit increased variability in size [8,9]. This change can be detected in the initial phase of infection, making MDW a useful marker for identifying sepsis before it progresses to more severe stages.

C-reactive protein (CRP) and procalcitonin (PCT) are well-established biomarkers used to detect infections and sepsis. However, in cancer patients, interpreting these biomarkers can be challenging due to the underlying malignancy and treatment-related factors that can also elevate CRP and PCT levels [10]. This study aimed to evaluate the diagnostic performance of MDW in identifying sepsis compared with other infection markers, including CRP and PCT, in patients who present to the emergency department (ED), particularly those with cancer. Furthermore, to assess the practical utility of MDW in real-world emergency settings, we compared its turnaround time (TAT) with that of conventional biomarkers.

## 2. Materials and Methods

### 2.1. Study Population

This retrospective study included patients who presented to the ED of a cancer hospital (Chonnam National University Hwasun Hospital, Hwasun, Republic of Korea) between 4 August 2020 and 31 March 2021. Patients aged 18–80 years who underwent CBC-DIFF testing using a DxH 900 analyzer (Beckman Coulter, Brea, CA, USA) were enrolled. Clinical data—including medical history, laboratory and microbiological findings, imaging test results, vital signs, ED management details, and hospitalization course—were collected from electronic medical records. The presence of malignancy, systemic inflammatory response syndrome (SIRS) criteria, quick Sequential Organ Failure Assessment (qSOFA), and Sequential Organ Failure Assessment (SOFA) scores were confirmed using hospital records.

### 2.2. Clinical Classification

The enrolled patients were categorized into three groups according to Sepsis-3 criteria [1]: control (patients with no evidence of infection), infection (patients with evidence of infection), and sepsis (patients with evidence of infection and ≥2 SOFA score). Baseline organ function was assumed to be normal for all patients. Patients were also classified into four groups based on the Sepsis-2 criteria [11,12]: control (patients with no evidence of infection and 0–1 SIRS criteria), SIRS (patients with no evidence of infection and ≥2 SIRS criteria), infection (patients with evidence of infection and 0–1 SIRS criteria), and sepsis (patients with evidence of infection and ≥2 SIRS criteria). Infection was confirmed by a positive culture result, or, when cultures were negative, by clinical diagnosis based on imaging findings and other clinical evidence.

Diagnostic performance was evaluated in all patients and further stratified into two subgroups based on the presence or absence of malignancy. Additional sub-analyses were performed for patients with leukopenia, defined as a white blood cell (WBC) count less than 4000/µL, and for those with suspected infection, identified by clinical signs such as ≥1 SIRS criteria, altered mental status, patient-reported dyspnea, or cases in which infection-focused diagnostics were initiated.

### 2.3. Laboratory Measurement

Blood samples were collected in K_3_ ethylenediaminetetraacetic acid (EDTA) tubes within 2 h and in serum separator tubes within 4 h of ED presentation, according to the intended analyses. CBC-DIFF and MDW analyses were performed within 1 h of sample collection using the DxH 900 analyzer. Neutrophil-to-lymphocyte ratio (NLR) and platelet-to-lymphocyte ratio (PLR) were calculated by dividing neutrophil and platelet counts by lymphocyte count, respectively. CRP was measured using the LABOSPECT 008AS (Hitachi, Tokyo, Japan) or Cobas 8000 (Roche, Basel, Switzerland), and PCT was assessed using the Cobas 8000 platform. TAT was defined as the interval from patient arrival at the ED to the time when the corresponding laboratory result was reported in the electronic medical record.

### 2.4. Statistical Analysis

For statistical analysis, the chi-square test was used for categorical variables, and the Kruskal–Wallis test was used for continuous variables. The Shapiro–Wilk test was employed to assess the assumption of normality. Diagnostic performance was evaluated by calculating the area under the receiver operating characteristic curve (AUC), sensitivity, specificity, positive predictive value, and negative predictive value (NPV). Two-sided 95% confidence intervals were calculated using the score method. Optimal cutoff values were determined using Youden’s index, optimized iteratively to maximize sensitivity and specificity. Comparisons between AUCs of different biomarkers were performed using the DeLong test. The paired *t*-test was used to compare TATs among the biomarkers. Statistical analyses were performed using Analyse-it (version 6.15.4, Analyse-it Software, Ltd., Leeds, UK) and Python (version 3.12.12, Python Software Foundation, Wilmington, DE, USA).

## 3. Results

### 3.1. Patient Characteristics

**Figure 1:**
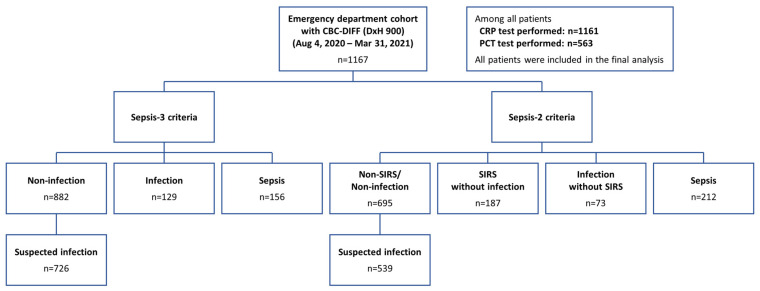
Flow diagram showing classification of all enrolled patients based on Sepsis-2 and Sepsis-3 definitions.

A total of 1167 patients who visited the emergency department were enrolled, and baseline characteristics of the overall population according to each Sepsis-3 and Sepsis-2 classification are presented in Figure 1, Table 1 and Table S1. The median age was 66, interquartile range (IQR) 57–73, and male patients accounted for 64.0% and 77.1% of patients had any history of malignancy. Among all patients with malignancies, 95.1% had solid tumors, while hematologic malignancies accounted for 4.9%.

According to Sepsis-3 criteria, 129 (11.1%) were classified as the infection group, and 156 (13.4%) as the sepsis group. The median qSOFA score was 0 (IQR 0–1) in both the infection and sepsis groups, and the median SOFA score was 1 (IQR 0–1) in the infection group and 4 (IQR 2.4–5) in the sepsis group. All inflammatory biomarkers were highest in the sepsis group.

Cultures of blood, sputum, urine, or other relevant specimens were performed in 667 patients (57.1%). Among the sepsis group according to Sepsis-3 criteria, 46.8% had positive cultures, 43.6% had negative cultures, and 9.6% had no cultures performed. The most frequently isolated pathogens were *Escherichia coli* and *Klebsiella pneumoniae*.

### 3.2. Diagnostic Performance of Biomarkers for Identification of Sepsis According to the Presence or Absence of Malignancy

**Figure 2:**
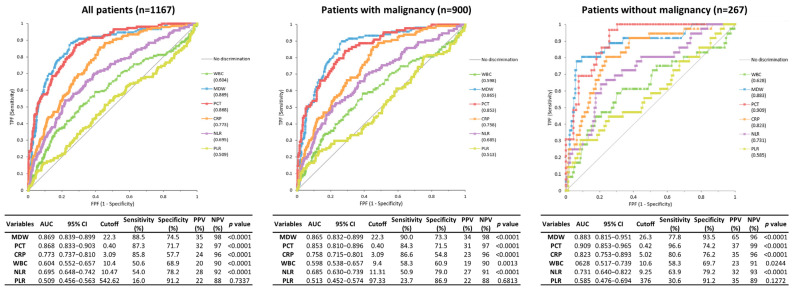
Receiver operating characteristic curves and diagnostic performance of six biomarkers for identification of sepsis (Sepsis-3 criteria) in three patient groups: all patients, patients with malignancy, and patients without malignancy.

In all patients, the discriminative performance of MDW for predicting sepsis appeared superior to that of WBC count, NLR, and PLR; slightly exceeded that of CRP; and was comparable to that of PCT according to Sepsis-3 criteria (Figure 2). MDW with a cutoff value of 22.3 showed the highest AUC (0.869), sensitivity (88.5%), and NPV (98%) among the biomarkers, followed by PCT (AUC of 0.868, sensitivity of 87.3%, NPV of 97% with cutoff value of 0.40 ng/mL) and CRP (AUC of 0.773, sensitivity of 85.8%, NPV of 96% with cutoff value of 3.09 mg/L). Analyses based on Sepsis-2 criteria showed consistent results (Figure S1).

In patients with malignancy, the diagnostic performance pattern of biomarkers for the identification of sepsis was similar to that of all patients. For the Sepsis-3 criteria, MDW showed an AUC of 0.865. Other biomarkers showed an AUC of 0.853 for PCT, 0.758 for CRP. In patients without malignancy, PCT demonstrated the best discriminative ability for sepsis, with an AUC of 0.909, followed by MDW (0.883) and CRP (0.823).

There was no statistically significant difference between MDW and PCT, whereas MDW showed a significantly higher AUC than CRP (*p* < 0.05) across all subgroups.

### 3.3. Diagnostic Performance of Biomarkers for Discrimination Between Infection and Non-Infection

**Figure 3:**
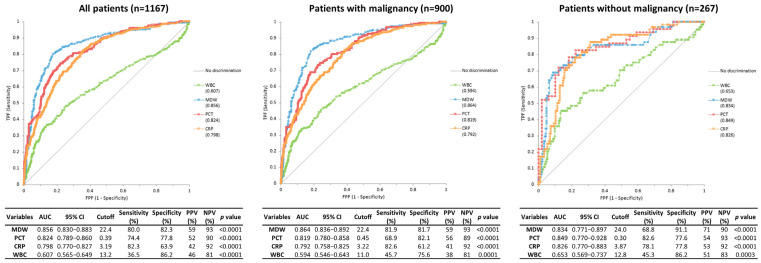
Receiver operating characteristic curves and diagnostic performance of four biomarkers for discrimination between infection and non-infection.

In all patients, MDW yielded the highest AUC for discrimination of infection (AUC 0.856), followed by PCT (AUC 0.824) and CRP (AUC 0.798) (Figure 3). In patients with malignancy, MDW showed the highest AUC (0.864), with PCT and CRP yielding AUCs of 0.819 and 0.792, respectively. In patients without malignancy, PCT achieved the highest AUC (0.849), with MDW (0.834) and CRP (0.826) following. Statistical comparison showed no significant difference between MDW and PCT, while MDW consistently outperformed CRP (*p* < 0.05).

### 3.4. Diagnostic Performance of Biomarkers for Identification of Sepsis in Patients with Leukopenia

In patients with leukopenia, the discrimination function of MDW to predict sepsis was not significantly different from that of PCT and CRP (Figure S2). MDW showed the highest AUC of 0.869, followed by CRP (AUC of 0.848) and PCT (AUC of 0.782).

### 3.5. Diagnostic Performance of Biomarkers for Identification of Sepsis and Infection in Patients with Initial Signs of Infection

**Figure 4:**
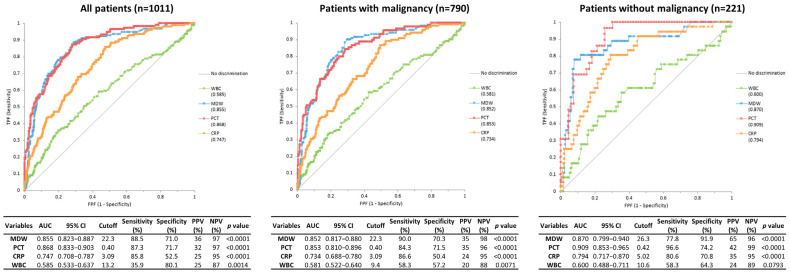
Receiver operating characteristic curves and diagnostic performance of four biomarkers for identification of sepsis (Sepsis-3 criteria) among patients with suspected infection.

In patients with initial signs of infection, PCT demonstrated the highest AUC for sepsis discrimination based on Sepsis-3 criteria (AUC 0.868), followed by MDW (0.855) and CRP (0.747) (Figure 4). Analyses based on Sepsis-2 criteria showed consistent trends (Figure S3). For infection discrimination, MDW yielded the highest AUC (0.843), followed by PCT (0.824) and CRP (0.775) (Figure S4).

In patients with malignancy, PCT and MDW showed comparable diagnostic accuracy for sepsis under Sepsis-3 criteria (AUCs of 0.853 and 0.852, respectively), whereas CRP yielded a lower AUC of 0.734. For infection identification, MDW demonstrated the highest AUC (0.853), followed by PCT (0.819) and CRP (0.771).

In patients without malignancy, PCT achieved the highest AUC for sepsis discrimination (AUC of 0.909), followed by MDW (0.870) and CRP (0.794). For infection identification, PCT again showed the best performance (AUC of 0.849), with MDW (0.816) and CRP (0.796) yielding slightly lower AUCs.

### 3.6. Screening Performance of Biomarkers with a Cutoff Value Recommended by the Manufacturer

According to Sepsis-3 criteria, when the cutoff value recommended by the manufacturer is applied to each inflammatory biomarker (21.5 for MDW, 0.30 mg/L for CRP, and 0.50 ng/mL for PCT), in non-infection patients, the proportions below the cutoff values for MDW, CRP, and PCT were 76.6%, 25.2%, and 84.0%, respectively (Table 2). In infection and sepsis patients, the proportions above the cutoff values for MDW, CRP, and PCT were 76.0%/91.0%, 94.5%/97.4%, and 48.3%/78.8%, respectively.

### 3.7. Comparison of TAT Among the Biomarkers

**Figure 5:**
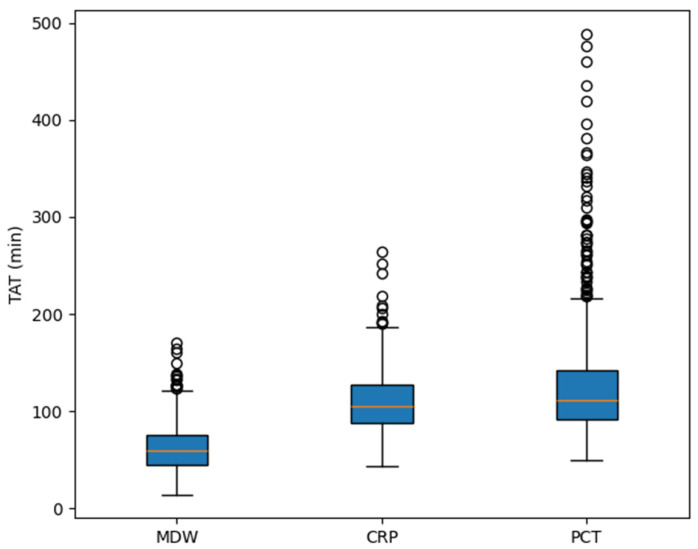
Comparison of turnaround time (TAT) among monocyte distribution width (MDW), C-reactive protein (CRP), and procalcitonin (PCT).

TATs for MDW, CRP, and PCT were compared in 561 patients who had all three measurements performed (Figure 5). The median TATs were 59 min, 105 min, and 111 min, respectively. MDW showed a significantly shorter TAT than both CRP and PCT (*p* < 0.0001 for both comparisons).

## 4. Discussion

Sepsis remains a formidable challenge in healthcare, contributing significantly to morbidity and mortality worldwide [13,14]. Prompt recognition and intervention are critical, as delays in diagnosis can lead to severe complications and increased mortality [2,3]. Recent advancements in diagnostic tools are paving the way for faster and more accurate detection of sepsis, thereby enhancing patient outcomes.

Approximately 20% of patients hospitalized with sepsis had a pre-existing cancer diagnosis, and this subgroup exhibited higher mortality compared to non-cancer patients [15]. These patients also present a particular diagnostic challenge, as malignancy-related conditions and therapy-induced systemic responses can mimic the clinical presentation of sepsis [4]. Furthermore, when sepsis does occur in cancer patients, it often presents atypically, which hinders timely recognition and diagnosis [10].

As diagnostic paradigms continue to evolve, the transition from Sepsis-2 to Sepsis-3 definitions has refined our understanding and classification of the condition. Sepsis-3, as proposed by Singer et al., emphasizes life-threatening organ dysfunction caused by a dysregulated host response to infection [1]. The SOFA and qSOFA scores are essential tools for evaluating the severity of sepsis, offering a standardized approach to assessing organ dysfunction and predicting patient outcomes.

However, despite the utility of qSOFA in predicting poor outcomes, accumulating evidence indicates that its limited sensitivity undermines its effectiveness as a standalone screening tool [16]. Our study also found that qSOFA had limited sensitivity in identifying patients with sepsis. Accordingly, exclusive reliance on qSOFA may delay sepsis recognition and compromise timely intervention, which has led to strong recommendations against its use as the sole screening method. In light of this, there is an urgent need for more sensitive and practical screening strategies that can facilitate early identification of sepsis.

MDW, an emerging biomarker, assesses monocyte heterogeneity by measuring the variability in monocyte size. This variability reflects the body’s inflammatory response, with elevated MDW levels indicating systemic inflammation and infection [8,9]. Notably, morphological changes in monocytes occur early in the inflammatory cascade, allowing MDW to detect immune activation at an early stage. By capturing these early alterations in monocyte size distribution, MDW offers valuable insight into the underlying inflammatory process and demonstrates strong potential as a practical indicator for the prompt identification of sepsis.

Multiple studies have supported the role of MDW in sepsis screening, particularly in emergency settings [6,7,17,18,19,20]. MDW offers several advantages, including a rapid turnaround time and widespread availability, as it is included in the routinely ordered CBC-DIFF [21]. This enables clinicians to access results promptly in most patients, supporting efficient and cost-effective clinical decision-making. Taken together, these characteristics support the feasibility of incorporating MDW into routine clinical workflows for sepsis evaluation.

CRP and PCT are commonly used biomarkers in sepsis diagnosis, but their specificity can be limited in patients with underlying conditions such as cancer, where elevated levels may result from non-infectious causes [10]. These limitations highlight the need for improved diagnostic strategies. The integration of multiple biomarkers, including MDW, CRP, and PCT, may enhance the diagnostic process, offering a more comprehensive approach to sepsis detection.

Consistent with previous studies [22,23,24,25,26,27], MDW demonstrated superior performance to CRP and comparable or slightly improved accuracy over PCT in diagnosing sepsis. Notably, this study expands those observations by showing that MDW maintains its diagnostic accuracy even in patients with malignancy, a subgroup often presenting diagnostic challenges. These results provide compelling evidence that MDW can be reliably used in patients with malignancy as well as the general population. Furthermore, MDW maintained consistent diagnostic accuracy in patients with leukopenia—a condition commonly observed in patients with malignancy, where reduced WBC counts may pose interpretive challenges for CBC-DIFF-derived parameters.

Beyond sepsis diagnosis, MDW also proved effective in differentiating between infectious and non-infectious cases, regardless of sepsis classification. Its performance remained strong not only across the entire cohort of emergency department patients but also within the subset clinically suspected of infection. This finding is particularly meaningful, as it reflects real-world practice where the selection of diagnostic tests is often based on clinical suspicion. These results support the utility of MDW as a broadly applicable and dependable biomarker for early detection of both infection and sepsis in acute care settings. Similar findings were reported in an independent cohort, further supporting the discriminatory ability of MDW between infectious and non-infectious conditions [28].

When evaluated using manufacturer-recommended cutoff values, MDW exhibited both higher specificity than CRP and greater sensitivity than PCT, positioning it as a biomarker with a balanced diagnostic profile. These findings underscore the distinct strengths of MDW, particularly in emergency settings where both timely recognition and diagnostic precision are essential. Nevertheless, as each biomarker captures distinct aspects of the host response, a multimodal approach involving MDW, CRP, and PCT may enhance diagnostic accuracy beyond what is achievable with any single marker. A recent systematic review reported that MDW, when applied as a standalone screening test in the ED, shows a very low false-negative rate and high NPV, highlighting its utility as a rule-out rather than a rule-in marker for sepsis [29]. Moreover, emerging data highlight the potential of MDW as part of a multimodal sepsis screening framework that integrates hematologic and biochemical markers to improve early detection accuracy [30,31].

In addition to its diagnostic accuracy, MDW demonstrated a markedly shorter TAT compared with CRP and PCT, with median values of 59 min, 105 min, and 111 min, respectively. This difference arises because MDW is automatically reported as part of the routine CBC-DIFF, making results available almost immediately after blood sampling. In contrast, CRP and PCT are not routine laboratory tests and must be specifically ordered when infection is clinically suspected, as they require separate biochemical assays. Consequently, their reporting is inherently delayed. Moreover, since MDW results can be available even before infection is clinically suspected, it may serve as an early alert marker that complements CRP and PCT. The rapid availability of MDW provides a significant clinical advantage in emergency settings, where timely information is essential for managing infection or sepsis.

This study has several limitations that warrant consideration. First, as a single-center retrospective analysis, the generalizability of the findings may be limited due to potential institutional or population-specific biases. The clinical practices, patient demographics, and laboratory procedures at the study site may not reflect those of other healthcare settings, thereby necessitating external validation across diverse centers. Second, baseline organ function was assumed to be normal for all patients when applying the Sepsis-3 criteria. Because the study population primarily consisted of patients with malignancy, some of whom may have had chronic hepatic or renal impairment related to the underlying disease or its treatment, this assumption should be interpreted with caution. It could have resulted in overestimation of organ dysfunction and, consequently, misclassification of sepsis severity, which might have affected biomarker performance. Another notable limitation lies in the potential variability in the timing of biomarker measurements. Differences in the temporal window for sample collection could influence diagnostic performance. To address this, the study included only MDW values obtained within 2 h and CRP/PCT values within 4 h of ED presentation, partially mitigating timing-related variability. Finally, cancer-related clinical heterogeneity and treatment factors were not specifically considered in this analysis. As these variables may influence monocyte activity and consequently MDW values, future prospective studies should incorporate detailed cancer characteristics and treatment histories to better elucidate their potential impact.

The findings of this study highlight the clinical utility of MDW as a rapid and accessible biomarker for early identification of sepsis and infection in emergency care settings. Its favorable performance relative to established markers such as CRP and PCT—especially in both the general population and in patients with malignancy—demonstrates its potential to enhance early clinical decision-making. Future research should explore the integration of MDW into clinical protocols and sepsis screening algorithms to assess its impact on patient outcomes in real-world settings.

## Figures and Tables

**Table 1:** Demographic and baseline characteristics of the patients according to Sepsis-3 criteria, medians (interquartile range).

Variables	Sepsis-3 Criteria			*p* Value
	Non-Infection (*n* = 882)	Infection (*n* = 129)	Sepsis (*n* = 156)	
Age, years	65.0 (55.9–72.0)	68.0 (58.0–74.0)	69.5 (61.4–75.6)	<0.0001
Sex				
Male, *n* (%)	574 (65.1%)	80 (62.0%)	93 (59.6%)	0.3737
Female, *n* (%)	308 (34.9%)	49 (38.0%)	63 (40.4%)	
Malignancy				
No, *n* (%)	203 (23.0%)	28 (21.7%)	36 (23.1%)	0.9448
Yes, *n* (%)	679 (77.0%)	101 (78.3%)	120 (76.9%)	
qSOFA score	0.0 (0.0–0.0)	0.0 (0.0–1.0)	0.0 (0.0–1.0)	0.0002
SOFA score	0.0 (0.0–2.0)	1.0 (0.0–1.0)	4.0 (2.4–5.0)	<0.0001
Culture				
Positive, *n* (%)	0 (0)	80 (62.0)	73 (46.8)	<0.0001
Negative, *n* (%)	409 (46.4)	37 (28.7)	68 (43.6)	
Not performed, *n* (%)	473 (53.6)	12 (9.3)	15 (9.6)	
WBC count (×10^9^/L)	8.20 (5.90–11.00)	10.40 (6.03–17.30)	10.55 (7.10–15.10)	<0.0001
CRP (mg/L)	1.30 (0.29–5.71)	9.35 (4.20–16.26)	9.54 (4.57–18.89)	<0.0001
PCT (ng/mL)	0.15 (0.07–0.34)	0.49 (0.18–0.98)	2.31 (0.58–9.39)	<0.0001
MDW	19.30 (17.60–21.30)	24.40 (21.60–26.93)	27.60 (24.14–32.10)	<0.0001

Abbreviations: qSOFA, quick Sequential Organ Failure Assessment; SOFA, Sequential Organ Failure Assessment; WBC, white blood cell; CRP, C-reactive protein; PCT, procalcitonin; MDW, monocyte distribution width.

**Table 2:** Patient distribution based on the cutoff value recommended by the manufacturer for MDW, CRP, and PCT in non-infection, infection, and sepsis groups (Sepsis-3 criteria).

Biomarkers	Cutoff	Non-Infection		Infection		Sepsis	
		Above	Less	Above	Less	Above	Less
MDW	21.5	23.4% (206/882)	76.6% (676/882)	76.0% (98/129)	24.0% (31/129)	91.0% (142/156)	9.0% (14/156)
CRP	0.30 mg/L	74.8% (657/878)	25.2% (221/878)	94.5% (121/128)	5.5% (7/128)	97.4% (151/155)	2.6% (4/155)
PCT	0.50 ng/mL	16.0% (57/356)	84.0% (299/356)	48.3% (43/89)	51.7% (46/89)	78.8% (93/118)	21.2% (25/118)

Abbreviations: MDW, monocyte distribution width; CRP, C-reactive protein; PCT, procalcitonin.

## Data Availability

The data presented in this study are available on request from the corresponding author due to ethical and institutional restrictions.

## Supplementary Materials

The following supporting information can be downloaded at https://www.mdpi.com/article/10.3390/jcm14228089/s1: Table S1: Demographic and baseline characteristics of the patients according to Sepsis-2 criteria, medians (interquartile range); Figure S1: Receiver operating characteristic curves and diagnostic performance of four biomarkers for identification of sepsis (Sepsis-2 criteria) in three patient groups: all patients, patients with malignancy, and patients without malignancy; Figure S2: Receiver operating characteristic curves and diagnostic performance of four biomarkers for identification of sepsis (Sepsis-3 criteria) in patients with leukopenia; Figure S3: Receiver operating characteristic curves and diagnostic performance of four biomarkers for identification of sepsis (Sepsis-2 criteria) among patients with suspected infection; Figure S4: Receiver operating characteristic curves and diagnostic performance of four biomarkers for discrimination between infection and non-infection among patients with suspected infection.
