# Supplementary material for: Diagnostic Utility of Monocyte Distribution Width for Early Sepsis Detection in Cancer-Enriched Emergency Cohort
Source: J Clin Med. 2025 Nov 14;14(22):8089. doi: 10.3390/jcm14228089 (PMC12653850; doi:10.3390/jcm14228089)
Supplement: Supplementary file 1 [file jcm-14-08089-s001.zip › jcm-3969515-supplementary.pdf]

Table S1. Demographic and baseline characteristics of the patients according to Sepsis-2 criteria, medians (interquartile range).

Variables	Sepsis-2 criteria				p value
	**Non-infection /Non-SIRS (n=695)**	**SIRS only (n=187)**	**Infection without SIRS (n=73)**	**Sepsis (n=212)**	
Age, years	66.0 (56.0–72.0)	64.0 (53.0–72.0)	67.0 (57.0–74.3)	69.0 (61.0–75.0)	<0.0001
Sex					
Male, n (%)	458 (65.9%)	116 (62.0%)	49 (67.1%)	124 (58.56)	0.2118
Female, n (%)	237 (34.1%)	71 (38.0%)	24 (32.9%)	88 (41.5%)	
Malignancy					
No, n (%)	168 (24.2%)	35 (18.7%)	18 (24.7%)	46 (21.7%)	0.4245
Yes, n (%)	527 (75.8%)	152 (81.3%)	55 (75.3%)	166 (78.3%)	
qSOFA score	0.0 (0.0–0.0)	0.0 (0.0–1.0)	0.0 (0.0–0.0)	0.0 (0.0–1.0)	<0.0001
SOFA score	0.0 (0.0–2.0)	1.0 (0.0–2.0)	1.0 (0.0–1.0)	3.0 (1.0–4.0)	<0.0001
Culture					
Positive, n (%)	0 (0)	0 (0)	45 (61.6)	108 (50.9)	<0.0001
Negative, n (%)	290 (41.7)	119 (63.6)	19 (26.0)	86 (40.6)	
Not performed, n (%)	405 (58.3)	68 (36.4)	9 (12.3)	18 (8.5)	
WBC count (x10^9/L)	7.80 (6.10–9.90)	12.70 (5.33–15.78)	9.20 (6.40–13.00)	11.10 (6.70–11.10)	<0.0001
CRP (mg/L)	1.00 (0.26–4.18)	5.03 (0.89–9.84)	8.15 (3.84–13.94)	9.74 (4.47–19.60)	<0.0001
PCT (ng/mL)	0.13 (0.06–0.32)	0.20 (0.09–0.41)	0.32 (0.15–0.69)	1.36 (0.49–5.78)	<0.0001
MDW	19.00 (17.40–20.90)	20.60 (18.61–23.06)	24.10 (20.90–26.86)	26.70 (23.40–31.10)	<0.0001

Abbreviations: SIRS, systemic inflammatory response syndrome; qSOFA, quick Sequential Organ Failure Assessment; SOFA, Sequential Organ Failure Assessment; WBC, white blood cell; CRP, C-reactive protein; PCT, procalcitonin; MDW, monocyte distribution width

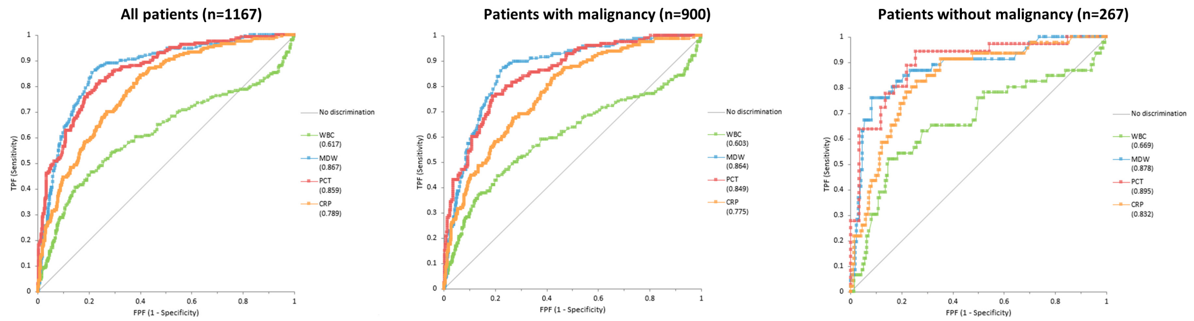

Variables	AUC	95% CI	Cutoff	Sensitivity (%)	Specificity (%)	PPV (%)	NPV (%)	*p* value
**MDW**	0.867	0.841–0.893	22.4	85.4	78.7	47	96	<0.0001
**PCT**	0.859	0.826–0.893	0.40	82.0	75.9	43	95	<0.0001
**CRP**	0.789	0.756–0.821	3.09	84.4	59.9	32	95	<0.0001
**WBC**	0.617	0.569–0.666	11.8	46.7	79.2	33	87	<0.0001

Variables	AUC	95% CI	Cutoff	Sensitivity (%)	Specificity (%)	PPV (%)	NPV (%)	*p* value
**MDW**	0.864	0.835–0.893	22.3	87.3	76.7	46	96	<0.0001
**PCT**	0.849	0.811–0.888	0.47	76.0	81.0	48	94	<0.0001
**CRP**	0.775	0.737–0.813	3.09	84.2	56.9	31	94	<0.0001
**WBC**	0.603	0.548–0.659	11.8	44.6	79.2	33	86	0.0003

Variables	AUC	95% CI	Cutoff	Sensitivity (%)	Specificity (%)	PPV (%)	NPV (%)	*p* value
**MDW**	0.878	0.818–0.938	24.9	76.1	91.9	66	95	<0.0001
**PCT**	0.895	0.826–0.963	0.30	94.4	74.6	44	98	<0.0001
**CRP**	0.832	0.770–0.894	3.87	82.6	74.2	40	95	<0.0001
**WBC**	0.669	0.570–0.767	12.8	52.2	85.1	42	90	0.0008

Figure S1. Receiver operating characteristic curves and diagnostic performance of four biomarkers for identification of sepsis (Sepsis-2 criteria) in three patient groups: all patients, patients with malignancy, and patients without malignancy.

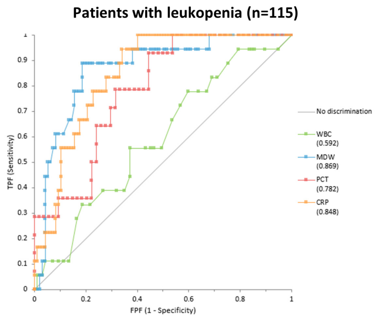

Variables	AUC	95% CI	Cutoff	Sensitivity (%)	Specificity (%)	PPV (%)	NPV (%)	*p* value
**MDW**	0.869	0.782–0.956	27.3	88.9	81.4	47	98	<0.0001
**PCT**	0.782	0.662–0.901	0.17	92.9	55.6	28	98	<0.0001
**CRP**	0.848	0.773–0.924	5.19	94.4	66.0	34	98	<0.0001
**WBC**	0.592	0.452–0.733	2.0	55.6	62.9	22	88	0.1986

Figure S2. Receiver operating characteristic curves and diagnostic performance of four biomarkers for identification of sepsis (Sepsis-3 criteria) in patients with leukopenia.

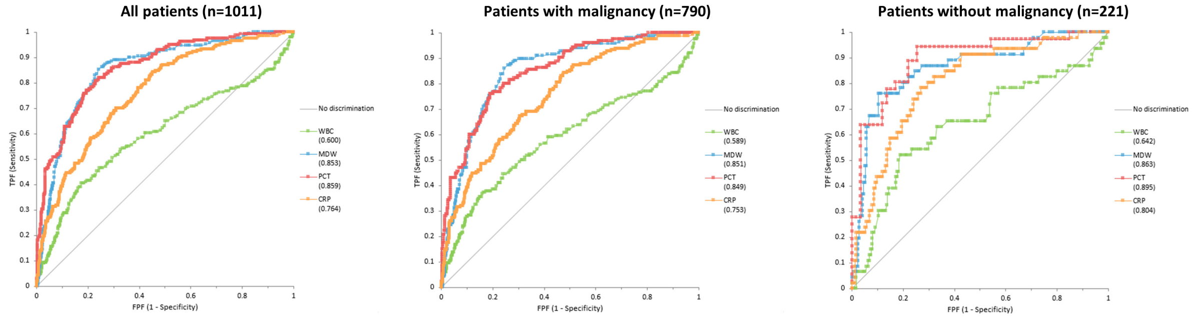

Variables	AUC	95% CI	Cutoff	Sensitivity (%)	Specificity (%)	PPV (%)	NPV (%)	*p* value
**MDW**	0.853	0.824–0.881	22.4	85.4	75.8	48	95	<0.0001
**PCT**	0.859	0.826–0.893	0.40	82.0	75.9	47	94	<0.0001
**CRP**	0.764	0.729–0.799	5.95	70.1	69.1	38	90	<0.0001
**WBC**	0.600	0.552–0.649	13.2	40.6	82.5	38	84	<0.0001

Variables	AUC	95% CI	Cutoff	Sensitivity (%)	Specificity (%)	PPV (%)	NPV (%)	*p* value
**MDW**	0.851	0.819–0.882	22.4	86.1	75.5	48	95	<0.0001
**PCT**	0.849	0.811–0.888	0.47	76.0	81.0	52	93	<0.0001
**CRP**	0.753	0.712–0.794	6.29	67.3	69.6	37	89	<0.0001
**WBC**	0. 589	0.534–0.644	13.4	37.3	83.7	38	83	0.0015

Variables	AUC	95% CI	Cutoff	Sensitivity (%)	Specificity (%)	PPV (%)	NPV (%)	*p* value
**MDW**	0.863	0.800–0.926	24.9	76.1	89.7	66	93	<0.0001
**PCT**	0.895	0.826–0.963	0.30	94.4	74.6	49	98	<0.0001
**CRP**	0.804	0.735–0.873	5.02	78.3	73.1	43	93	<0.0001
**WBC**	0.642	0.543–0.742	12.8	52.2	81.1	42	87	0.0051

Figure S3. Receiver operating characteristic curves and diagnostic performance of four biomarkers for identification of sepsis (Sepsis-2 criteria) among patients with suspected infection.

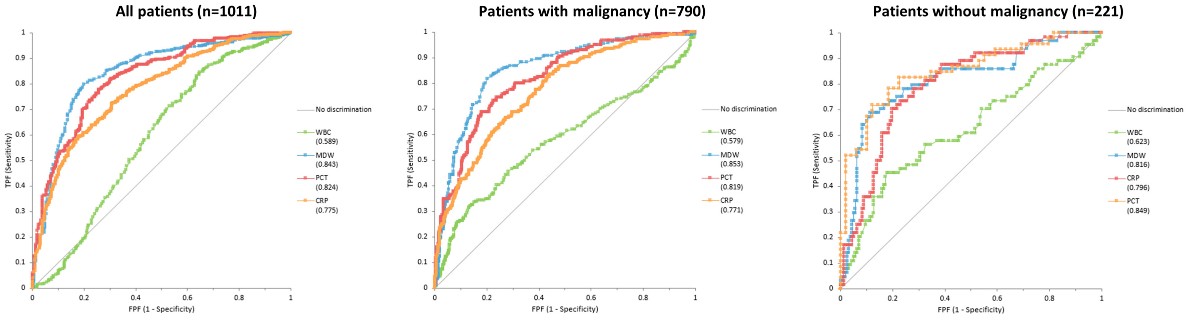

Variables	AUC	95% CI	Cutoff	Sensitivity (%)	Specificity (%)	PPV (%)	NPV (%)	*p* value
**MDW**	0.843	0.814–0.871	22.5	79.9	80.0	91	61	<0.0001
**PCT**	0.824	0.789–0.860	0.39	77.8	74.4	89	57	<0.0001
**CRP**	0.775	0.744–0.806	5.93	72.4	69.3	86	50	<0.0001
**WBC**	0.589	0.547–0.631	13.3	83.2	36.5	77	46	<0.0001

Variables	AUC	95% CI	Cutoff	Sensitivity (%)	Specificity (%)	PPV (%)	NPV (%)	*p* value
**MDW**	0.853	0.823–0.883	22.4	81.9	79.8	61	92	<0.0001
**PCT**	0.819	0.780–0.858	0.45	68.9	82.1	60	87	<0.0001
**CRP**	0.771	0.735–0.807	6.29	66.7	72.8	49	85	<0.0001
**WBC**	0.579	0.531–0.627	14.0	32.1	86.5	48	77	0.0014

Variables	AUC	95% CI	Cutoff	Sensitivity (%)	Specificity (%)	PPV (%)	NPV (%)	*p* value
**MDW**	0.816	0.750–0.883	24.0	68.8	88.5	71	87	<0.0001
**PCT**	0. 849	0.770–0.928	0.30	82.6	77.6	60	92	<0.0001
**CRP**	0.796	0.733–0.859	5.86	70.3	80.3	59	87	<0.0001
**WBC**	0.623	0.537–0.709	12.8	45.3	82.2	51	79	0.0051

Figure S4. Receiver operating characteristic curves and diagnostic performance of four biomarkers for discrimination between infection and non-infection among patients with suspected infection.
